# Musical auditory training and cognitive auditory training: effects on the perception of tinnitus disorder

**DOI:** 10.1590/2317-1782/e20250145en

**Published:** 2026-04-03

**Authors:** Christine Grellmann Schumacher, Brenda Larissa de Souza da Silva, Roberta Liberalesso, Katya Guglielmi Marcondes Freire, Michele Vargas Garcia, Dayane Domeneghini Didoné

**Affiliations:** 1 Departamento de Fonoaudiologia, Centro de Ciências da Saúde, Universidade Federal de Santa Maria – UFSM - Santa Maria (RS), Brasil.

**Keywords:** Tinnitus, Auditory Perception, Hearing, Controlled Clinical Trial, Evoked Potentials, Auditory

## Abstract

**Purpose:**

To examine the effects of Cognitive Auditory Training (CAT) and Musical Auditory Training (MAT) on the self-perception of adults with tinnitus.

**Methods:**

Preliminary comparative clinical, interventional and longitudinal study, approved by the Ethics Committee of Research at Federal University of Santa Maria under protocol number 5.746.241. The study included 29 adults of both sexes with tinnitus and normal results in a basic audiological evaluation. Participants were divided into three groups: 13 individuals in the CAT group, eight in the MAT group, and eight in the placebo intervention group. Scores for Disturbance and Loudness on the Visual Analog Scale (VAS D and VAS L) and the Tinnitus Handicap Inventory (THI) were analyzed before and after the interventions. The Wilcoxon test was used for pre- and post-intervention comparisons, with a significance level of 5%.

**Results:**

In the CAT group, statistically significant differences were observed for VAS D, VAS L, and THI. In the MAT group, significant differences were found for VAS D and THI. In the placebo group, statistically significant differences were observed for VAS D and THI.

**Conclusion:**

Improvements in symptom perception were observed across all three groups. However, CAT demonstrated greater effectiveness, showing improvements in all assessed aspects.

## INTRODUCTION

Research on the effectiveness of different interventions for tinnitus is important, given the scarcity of different treatment protocols aimed at reorganizing auditory and non-auditory brain areas and reducing the perception of the symptom^(1)^.

Tinnitus is directly related to alterations in neuronal activity in central auditory pathways^(2,3)^. The neurobiological basis of this symptom is defined by a continuous abnormal spontaneous activity, characterized by the disorganization of neural pathways of the central auditory system^(3)^. In addition, there is a disorganization of non-auditory brain areas, including the frontoparietal area responsible for consciousness and attention; the neural network related to emotions, which consists of the anterior cingulate cortex, anterior insula, and amygdala; the hippocampus and the parahippocampal area, which reflect the activity of memory and cognition mechanisms, which are also associated with the persistence of tinnitus perception, generating anxiety, distress, and suffering^(2)^.

Tinnitus is a multifactorial auditory symptom, characterized by the perception of a sound in the absence of external acoustic stimuli, with the individual’s perception occurring in the head or ears^(1)^. It is estimated that this symptom affects approximately 21% of the adult population, and that 1% to 3% of individuals present a significant impact on their daily activities^(4,5)^.

The identification of suffering resulting from tinnitus perception leads to the definition of the term “tinnitus disorder,” which can be described as the conscious perception of a tonal or composite sound, associated with emotional dysfunction, cognitive alteration, and/or autonomic arousal, leading to behavioral changes and functional disability^(6)^. In addition to this impact on daily activities, the literature reports the repercussions of the symptom on social, emotional, and professional activities, which causes a negative impact on the quality of life of these individuals and, consequently, greater perception of and annoyance with tinnitus^(7)^.

Sadeghijam et al.^(8)^ described the chaos theory, in which tinnitus disorder is a resulting factor of changes in the dynamic and nonlinear functioning of the central auditory system. Thus, any neural alteration resulting from an imbalance between excitability and the inhibition mechanism or from a reduction in auditory input will result in a compensatory mechanism, converting into an amplification of spontaneous and synchronous neural activity^(8)^.

One of the alternatives to reorganize auditory neural pathways is through auditory training^(9,10)^, characterized as a form of therapy composed of a set of tasks that seek to reorganize the functioning of the central auditory system through positive neuroplasticity. Auditory neuroplasticity is the ability of the central nervous system to reorganize and adapt its neural connections in response to sound stimuli and auditory experiences, promoting functional changes in areas related to hearing^(11)^.

Individuals with tinnitus disorder may present neuroplastic dysfunction due to deafferentation of central regions^(12,13)^, and these alterations can be reestablished with auditory training, which reorganizes the central auditory pathway through acoustic exercises that promote greater neural synchrony due to the mechanism of neuroplasticity^(1)^.

Researchers^(1,14)^ describe that auditory training may be an option for reducing tinnitus perception and annoyance. Furthermore, auditory-cognitive tasks are described as essential in cases of tinnitus, as pointed out by a systematic review^(1)^, which identified that attentional and multisensory factors showed improved outcomes.

There are different proposals for auditory training, one of which is Musical Auditory Training (MAT), defined as an instrument that can generate changes in auditory pathways, both structural and functional, and an improvement in auditory skills and individual performance in response to different acoustic events, being related to the capacity for reorganization of neural pathways^(15)^. Scientific evidence demonstrates that this is an intervention to be performed when there are alterations in auditory processing; however, this technique may promote the neural reorganization necessary in tinnitus disorder^(15)^. Nevertheless, there are still no studies that prove its effectiveness regarding symptom perception in the population with tinnitus disorder.

Cognitive Auditory Training (CAT), in turn, may be a proposed intervention to reduce tinnitus perception, as it aims to stimulate auditory and cognitive skills, such as attention, memory, figure–ground for verbal sounds, temporal ordering and resolution, auditory closure, executive functions, and motor praxis^(16)^. Studies have shown that stimulating cognitive skills in cases of tinnitus disorder may be essential for reducing symptom perception, since the consequences of tinnitus in the central nervous system involve a plastic reorganization that encompasses auditory and non-auditory areas of the central nervous system, and multisensory perceptual learning tends to be more consistent than unimodal learning^(17)^.

Thus, in an attempt to contribute to the scientific gap regarding the limited forms of intervention and their effectiveness for tinnitus disorder, the present study sought to verify the effects of Cognitive Auditory Training (CAT) and Musical Auditory Training (MAT) on the self-perception of adult individuals with tinnitus disorder.

## METHOD

This is a preliminary, comparative, interventional, longitudinal clinical study. The study was approved by the Research Ethics Committee of the Federal University of Santa Maria, under opinion no. 5.746.241. Before the beginning of the research, all participants signed the Free and Informed Consent Form (ICF). The study was conducted at the Audiology Outpatient Clinic of the aforementioned University. The research was disseminated on social media, and participant selection occurred in a random, non-probabilistic manner.

During the participant recruitment phase, the following eligibility criteria were established: individuals of both sexes, aged between 18 and 55 years; complaint of unilateral or bilateral subjective tinnitus disorder, with symptom perception for at least six months^(18)^; not undergoing intervention or pharmacological treatment for tinnitus; and absence of a diagnosis of neurological or psychiatric diseases. For participants who met the recruitment criteria, the following inclusion criteria were established:

- Hearing thresholds within normal limits or mild sensorineural hearing loss according to the World Health Organization^(19)^;
- Type A tympanometric curve, according to the Jerger classification^(20,21)^;
- Presence of contralateral stapedial acoustic reflexes at normal levels^(22,23)^;
  ● Visual Analog Scale (VAS) annoyance score of at least 4, indicating moderate symptom annoyance, pre-intervention^(24)^.

The following exclusion criteria were considered:

- Failure to complete any of the stages proposed for the study.

### Sample composition procedures

Initially, volunteers underwent anamnesis based on the *Clinical Practice Guideline: Tinnitus* ^(25,26)^ to understand their clinical history. Participants were questioned about hearing, previous history of tinnitus, and neurological impairment or psychiatric treatments.

Subsequently, visual inspection of the external auditory canal was performed using a Mikatos otoscope, with the aim of verifying possible impediments to the performance of the other audiological procedures.

All volunteers underwent basic audiological evaluation, consisting of pure-tone audiometry and speech audiometry, in an acoustically treated booth, using an Interacoustics AD229e audiometer and TDH-39 supra-aural headphones from Telephonics.

Air-conduction hearing thresholds were investigated at frequencies from 250Hz to 8kHz, bilaterally, using the descending–ascending technique. Thresholds below 20dBHL were considered normal, according to the quadritonal average of 500Hz, 1kHz, 2kHz, and 4kHz, as proposed by the WHO^(19)^.

Acoustic immittance measures were performed using the Interacoustics AT 235 equipment, with TDH-39 headphones from Telephonics. Tympanometric curves and contralateral stapedial acoustic reflexes (frequencies of 500, 1000, 2000, and 4000Hz) were investigated to assess the integrity of the middle ear and the reflex arc. Analysis of tympanometric curve results was conducted according to Jerger, Jerger, and Mauldin^(21)^, and contralateral stapedial acoustic reflexes according to Gelfand^(22)^ and Jerger and Jerger^(23)^.

### Research procedures

Following the audiological evaluations described above, participants underwent assessment of tinnitus annoyance and loudness measurement. It should be noted that all equipment used was calibrated in March 2023, coinciding with the data collection period of the present study. The Visual Analog Scale (VAS) and the Tinnitus Handicap Inventory (THI) were used for this purpose.

The analysis of self-perception of tinnitus disorder is an important factor, constituting a complementary approach to measure the impact of tinnitus on the patient’s life pre- and post-intervention^(27)^. There are several instruments that can be used to measure and monitor the individual’s perception of the symptom, among which the Visual Analog Scale (VAS) and the Tinnitus Handicap Inventory (THI) questionnaire stand out^(28)^.

Thus, it is possible to subjectively measure the level of discomfort of participants regarding the tinnitus symptom. Both procedures, THI and VAS, were used in the evaluation and two weeks after completion of the intervention. Quantification of the degree of annoyance and loudness was defined using the VAS, a self-perception scale presented through a figure numbered from zero to ten. Volunteers were asked about their subjective perception of the degree of annoyance and loudness and instructed to indicate the number that best matched their perception, with zero representing no annoyance and tinnitus without loudness, and ten representing severe annoyance and very loud tinnitus^(24)^. Furthermore, the inclusion criterion of a score of at least four points indicating moderate annoyance characterizes the individual’s perception as tinnitus disorder.

The THI is a questionnaire composed of 25 questions related to the individual’s social and personal life, which are directly associated with the negative changes caused by tinnitus in daily life and its interference with the quality of life of these individuals. The questions were administered orally, with possible responses of “No,” “Sometimes,” and “Yes,” scored as zero, two, and four points, respectively. To measure symptom annoyance, the sum of the questions was used, with lower values indicating lower perceived annoyance^(20)^.

### Intervention procedures

Regarding intervention procedures, participants were randomly allocated into three groups according to the order of arrival for care: the CAT Group, which received intervention through CAT; the MAT Group, which received intervention through MAT; and the placebo Group, which received placebo intervention. All interventions were conducted in person and administered by a researcher.

For CAT, the protocol proposed by Schumacher et al.^(29)^ was used, and six sessions of auditory and cognitive skills training were applied weekly, totaling six weeks, that is, one session per week. Each session lasted 45 to 50 minutes. Throughout the intervention, all strategies aimed to stimulate auditory skills and cognitive aspects of participants, such as: figure–ground for verbal sounds and selective attention; attention; temporal ordering for duration; selective attention, executive functions, motor praxis; auditory discrimination for non-verbal sounds; constructive praxis; language; discourse processing; temporal ordering for frequency; and memory. Chart 1 presents the CAT protocol used in the present study.

**Chart 1:** Cognitive Auditory Training protocol used in the present study, considering the auditory and cognitive skills stimulated in each session

**Session**	**Stimulated Skill**
1º	- Attention;
	- Auditory: figure–ground for verbal sounds and selective attention;
	- Memory;
	- Temporal ordering for duration;
	- Temporal ordering;
2º	- Attention;
	- Executive functions;
	- Motor praxis;
	- Memory;
	- Discrimination;
	- Auditory discrimination for non-verbal sounds;
3º	- Attention;
	- Memory;
	- Executive functions;
	- Constructive praxis;
	- Auditory discrimination for non-verbal sounds;
4º	- Language;
	- Memory;
	- Figure–ground for verbal sounds and selective attention;
	- Attention;
	- Discourse processing;
	- Temporal ordering for frequency;
	- Auditory discrimination for non-verbal sounds;
5º	- Figure–ground for verbal sounds;
	- Attention;
	- Auditory discrimination;
	- Memory;
	- Auditory ordering;
	- Auditory closure;
6º	- Memory;
	- Temporal ordering;
	- Temporal resolution;

The protocol used for the application of MAT was proposed by Freire^(15)^. Eight sessions were conducted, with duration ranging from 45 to 50 minutes per session. During this four-week period, auditory skills such as figure–ground for instrumental sounds, figure–ground for sequential sounds, directed listening, temporal ordering for duration and frequency, rhythm, auditory closure, and audiovisual memory were stimulated. At the beginning of each session, participants were instructed about the activities to be performed that day. Auditory training was conducted using supra-aural headphones at an acoustically comfortable intensity. Chart 2 specifies the MAT protocol used in the present study.

**Chart 2:** Musical Auditory Training protocol used in the present study, considering the auditory skills stimulated in each session

**Session**	**Stimulated Skill**
1º	Figure–ground for instrumental sounds
2º	Figure–ground for sequential sounds
3º	Directed listening
4º	Temporal ordering for duration
5º	Temporal ordering for frequency
6º	Rhythm
7º	Auditory closure
8º	Audiovisual memory

The protocol used for the placebo group consisted of exposure to films and classical music presented in random order across different sessions. Participants were instructed to pay visual attention to the film (presented without sound) and auditory attention to the music. The films presented were: *Cirque Du Soleil*, entitled “Journey of Man”; the Chaplin Collection, including the films “Modern Times,” “The Great Dictator,” “The Gold Rush,” and “Limelight.” It is noteworthy that the selected films were cinematic works that do not depend on dialogue for viewer understanding. The music presented was *Sonata for Two Pianos in D Major, K448*, by Mozart, without masking the tinnitus loudness. The placebo group protocol consisted of eight sessions, conducted twice a week, lasting at least 45 to 50 minutes.

Two weeks after completion of the interventions, participants again completed the THI and VAS questionnaires to measure possible changes in symptom perception following the interventions performed. THI and VAS results were compared according to the distribution of individuals among the described groups.

A total of 95 individuals applied to participate in the research. However, 11 individuals were not recruited due to the presence of previously diagnosed neurological and/or psychiatric diseases, and 13 due to already being in some form of tinnitus treatment. Of the 71 individuals who underwent evaluations for sample composition, 14 were excluded due to otitis and/or Eustachian tube dysfunction, and 18 due to moderate to profound hearing loss. Additionally, 10 individuals were excluded for not completing all stages of the study.

Thus, the final sample consisted of 29 individuals randomly distributed among the three groups. The groups were composed of 13 individuals in the CAT group, 8 in the MAT group, and 8 in the placebo group. The difference in participant distribution among groups occurred due to loss to follow-up in the groups that received intervention through MAT and placebo.

A double-blind design was adopted for the study, in which participants were unaware of which intervention model they were receiving, and a different researcher was assigned to each stage of the study: assessment, intervention, and rehabilitation. After the placebo intervention, follow-up treatment was offered to participants through MAT or CAT, according to the participant’s choice based on their affinity with each intervention proposal.

Data related to the VAS scale and the THI questionnaire pre- and post-intervention were entered into an Excel spreadsheet, and statistical analyses were performed using SPSS software, version 30.0, by a professional in the field. Initially, the Shapiro–Wilk test was used to verify data normality and, consequently, to select the statistical test. Intergroup analysis was performed using the Kruskal–Wallis test, and in cases of statistically significant differences, data were compared in pairs. Pairwise comparisons were performed using the Bonferroni post-hoc test. Intragroup comparisons were performed using the Wilcoxon test. A significance level of 5% was adopted for all analyses performed.

## RESULTS

The groups were considered equivalent through statistical comparison, with no significance. The study sample consisted of three groups with the following sex distributions: CAT (6 men and 7 women), MAT (3 men and 5 women), and placebo group (3 men and 5 women), with no statistically significant difference among them (p-value = 0.333), according to Fisher’s exact test analysis. The mean level of education, expressed in years of schooling, was 12.69 (minimum of 12 and maximum of 15) in the CAT group, 13.5 (minimum of 12 and maximum of 15) in the MAT group, and 13.13 (minimum of 12 and maximum of 15) in the placebo group, with no statistically significant difference (p-value = 0.454), according to the Kruskal–Wallis test. The mean age was 29.64 years (minimum of 22 and maximum of 49) in the CAT group, 37 years (minimum of 22 and maximum of 53) in the MAT group, and 31 years (minimum of 20 and maximum of 53) in the placebo group, also with no statistically significant difference among the groups (p-value = 0.379), according to the Kruskal–Wallis test.

The data from the VAS annoyance (VAS A), VAS loudness (VAS L), and pre-intervention THI were compared among the groups. Homogeneity among the groups was observed for VAS L and VAS A (Table 1). However, comparison among the three groups showed differences for THI (p = 0.006). Pairwise comparison showed differences between the MAT and placebo groups (p = 0.004) for pre-intervention THI, with lower values for the MAT group. Comparisons between MAT and CAT (p = 0.344) and between CAT and placebo (p = 0.143) showed no statistically significant differences.

**Table 1:** Comparison of VAS L, VAS A, and pre-intervention THI among the groups

	**Group**	**Minimum**	**Maximum**	**Mean**	**Median**	**P-value**
VAS A pre	MAT	4	9	7	7.5	0.900
	CAT	3	10	6.85	8	
	Placebo	4	10	6.38	6.50	
	MAT	5	8	6.63	7	0.560
VAS L pre	CAT	4	10	7.15	8	
	Placebo	4	9	6.38	6.50	
	MAT	20	58	34	31	0.006
THI pre	CAT	22	80	47.23	40	
	Placebo	36	94	66	66	

**Caption:** VAS A: Visual Analog Scale for Annoyance; VAS L: Visual Analog Scale for Loudness; THI: Tinnitus Handicap Inventory. For statistical analysis, the Kruskal-Wallis test was used

The data from VAS A, VAS L, and THI were also compared among the groups post-intervention (Table 2), and a statistically significant difference was found for all variables among the three groups.

**Table 2:** Comparison of VAS A, VAS L, and post-intervention THI among the groups

	**Group**	**Minimum**	**Maximum**	**Mean**	**Median**	**P-value**
VAS A post	MAT	1	8	4.38	4.50	0.040
	CAT	0	7	2.77	3.00	
	Placebo	1	8	4.38	4.50	
	MAT	3	8	5.63	5.50	0.003
VAS L post	CAT	0	6	2.92	3.00	
	Placebo	3	8	5.63	5.50	
	MAT	2	32	18.25	18.00	0.015
THI post	CAT	4	65	21	16.00	
	Placebo	19	58	39.38	39.00	

**Caption:** VAS A: Visual Analog Scale for Annoyance; VAS L: Visual Analog Scale for Loudness; THI: Tinnitus Handicap Inventory. For statistical analysis, the Kruskal-Wallis test was used

Pairwise comparison showed differences for VAS A (p = 0.050) and VAS L (p = 0.021) between the CAT and placebo groups, with better results for the CAT group, evidenced by a greater reduction in values. For VAS A, the other post-hoc comparisons showed no differences (CAT and MAT, p = 0.195; MAT and placebo, p = 1.000).

For the VAS L scale, a statistically significant difference was also observed between the MAT and CAT groups (pairwise method) (p = 0.011) and between CAT and placebo (p = 0.021), with better results for the CAT group (Table 2). The comparison between CAT and MAT showed no differences (p = 1.000).

For the THI questionnaire, a statistically significant difference was observed in the comparison among the three groups in the post-intervention moment (p = 0.015). Pairwise analysis showed differences between the MAT and placebo groups (p = 0.047), with better results for the MAT group, and differences between CAT and placebo (p = 0.023), with better results for the CAT group. The comparison between CAT and MAT showed no statistically significant difference (p = 1.000) (Table 2).

Considering intragroup comparisons, Table 3 shows the values of the VAS annoyance (VAS A), VAS loudness (VAS L), and THI pre- and post-intervention for the CAT group. A statistically significant difference was observed for all analyzed variables, with better results after the CAT intervention.

**Table 3:** Data from the VAS for annoyance and loudness and total THI questionnaire score for the group that received the intervention through CAT

	**Mean**	**Median**	**Minimum**	**Maximum**	**SD**	**P-value**
VAS A pre	6.85	8.00	4	10	2.23	0.002
VAS A post	2.77	3.00	0	7	1.64	
VAS L pre	7.15	8.00	4	10	2.11	0.002
VAS L post	2.92	3.00	0	6	1.55	
THI pre	47.23	40.00	22	80	18.61	0.001
THI post	21.00	16.00	4	65	17.95	

**Caption:** VAS A: Visual Analog Scale for Annoyance; VAS L: Visual Analog Scale for Loudness; THI: Tinnitus Handicap Inventory; SD: standard deviation. For statistical analysis, the Wilcoxon test was used

Table 4 presents the values for the group that received MAT, with statistically significant differences for VAS A and THI, with all results being better after the MAT intervention.

**Table 4:** Data from the VAS for annoyance and loudness and total THI questionnaire score for the group that received the intervention through MAT

	**Mean**	**Median**	**Minimum**	**Maximum**	**SD**	**P-value**
VAS A pre	7.00	7.50	4	9	1.85	0.028
VAS A post	4.38	4.50	1	8	2.13	
VAS L pre	6.63	7.00	5	8	0.91	0.102
VAS L post	5.35	5.50	3	8	1.68	
THI pre	34.00	31.00	20	58	13.93	0.001
THI post	18.25	18.00	2	32	11.73	

**Caption:** VAS A: Visual Analog Scale for Annoyance; VAS L: Visual Analog Scale for Loudness; THI: Tinnitus Handicap Inventory; SD: standard deviation. For statistical analysis, the Wilcoxon test was used

Table 5 presents the pre- and post-intervention results for the placebo group, with statistically significant differences observed for VAS A and THI. All results indicate better scale values in the post-intervention period.

**Table 5:** Data from the VAS for annoyance and loudness and total THI questionnaire score for the group that received the placebo intervention

	**Mean**	**Median**	**Minimum**	**Maximum**	**SD**	**P-value**
VAS A pre	6.62	6.5	4	10	2.13	0.027
VAS A post	4.88	5.0	1	8	2.29	
VAS L pre	6.38	6.5	4	9	1.68	0.053
VAS L post	5.50	5.5	3	8	2.07	
THI pre	66.00	66.00	36	94	16.21	0.012
THI post	39.38	39.00	19	58	14.41	

**Caption:** VAS A: Visual Analog Scale for Annoyance; VAS L: Visual Analog Scale for Loudness; THI: Tinnitus Handicap Inventory; SD: standard deviation. For statistical analysis, the Wilcoxon test was used

## DISCUSSION

This study addressed two types of intervention through the stimulation of auditory skills, which were combined with cognitive and musical interventions through CAT, MAT, and a placebo group, respectively. When analyzed, these interventions demonstrated an improvement in the perception of annoyance (VAS A) and quality of life (THI), suggesting that all proposed interventions modified symptom perception.

In the analysis of pre-intervention data, it was possible to verify that the data were homogeneous for VAS A and VAS L across all groups. Considering the analysis of post-intervention data, it was observed that the results of the CAT group were better when compared with the MAT and placebo groups. These results corroborate a recent study^(29)^ that also demonstrated improvement in the perception of tinnitus disorder through CAT, suggesting that auditory and cognitive stimulation promotes benefits in symptom perception.

For the THI questionnaire, differences between groups in the post-intervention phase were observed between the MAT and placebo groups, with better results for the MAT group. However, these results should be interpreted with caution, since THI data for these groups were not considered homogeneous in the pre-intervention stage. Because this is a preliminary study, it should be considered that these pre-intervention differences may be related to the reduced sample size. Despite this, these results are promising, given that MAT is a current approach and allows reorganization of the auditory pathway of these individuals and improvement in symptom perception. It should be emphasized that no studies were found in the scientific literature to compare these data with previous findings.

Differences in post-intervention THI were also observed between the CAT and placebo groups, with better results for the CAT group. Cognitive auditory intervention has been shown to be a promising alternative for tinnitus treatment, as it promotes reorganization of auditory pathways associated with cognition^(16,30)^.

The intragroup comparisons in this study, presented in Tables 3, 4, and 5, are in line with a recently published scoping review^(1)^, which identified 15 studies with auditory and cognitive training approaches. Among these, only four did not find statistically significant effects after intervention. This suggests that auditory training, in its different approaches, is a rehabilitation option for tinnitus disorder.

It was observed that intervention with CAT (Table 3) led to significant improvements in VAS A, VAS L, and THI scores, indicating its effectiveness in treating tinnitus perception. These findings are consistent with studies that employed cognitive training^(30)^ and observed improvements in questionnaires assessing symptom perception. This benefit may be associated with the central stimulation promoted by this training, which stimulates neuroplasticity of auditory and cognitive functions. This occurs because tinnitus induces plastic reorganization in the central nervous system, encompassing both auditory and non-auditory areas, and multisensory perceptual learning proves to be more durable and consistent compared with unimodal learning^(1)^.

In addition, in a study on cognitive therapy, including Heidelberg Neuro-Music Therapy applied by Krick et al.^(31)^, it was observed that the treatment helps patients with chronic tinnitus shift their attention from phantom auditory perception to visual stimuli. This change in focus may involve the angular gyrus, an essential brain area for orienting attention toward more meaningful auditory stimuli. This finding strengthens the basis for CAT as a rehabilitation option, as it provides auditory and cognitive stimulation.

Regarding the findings of MAT (Table 4), improvements were obtained in VAS A scores and the THI questionnaire. No studies were found regarding the effect of MAT in patients with tinnitus disorder, which confers novelty to this intervention in the population of the present study. However, the benefits observed in the present research are consistent with another study involving older adults who were users of sound amplification^(32)^. Although the cited study was conducted with older adults with hearing loss and users of electronic sound amplification devices, the authors verified benefits of MAT on quality of life, depressive symptoms, cognitive aspects, temporal resolution, and limitations in activities of daily living. Improvement in these symptoms after MAT stimulation may be associated with the results of the present study, since the positive impact of MAT on the aforementioned aspects influences tinnitus perception. This may be reflected in improved quality of life and reduced symptom annoyance reported by the participants of this study.

Despite the satisfactory results of MAT intervention, no statistically significant changes were observed for the VAS related to loudness, suggesting that loudness did not interfere with the quality of life of the studied sample. It may also be considered that loudness perception did not improve due to the fact that MAT does not stimulate cognitive skills as strongly as CAT, since stimulation is focused on musical perception and auditory skills. As previously mentioned by other studies^(33,34)^, multisensory interventions seem to be more effective in reducing symptom perception, which justifies the findings described.

In this study, the placebo intervention also showed significant improvements in THI and VAS A scores, highlighting the relevance of patient support, favored by in-person contact and direct interaction with participants during the sessions. It should be emphasized that this intervention was conducted in a double-blind format, ensuring that participants were unaware of whether they were receiving active treatment or placebo. Additional studies support the beneficial effects of placebo intervention, associating empathetic support with attenuation of reported symptoms^(35-37)^.

The literature highlights that the support provided during placebo intervention and a longitudinal intervention play crucial roles in the treatment of patients with tinnitus disorder, contributing to a more effective and humanized therapeutic approach. Support promotes a safe and trusting environment in which the patient feels understood and supported, which may reduce tinnitus-related stress and improve treatment adherence. A longitudinal intervention, in turn, allows continuous and adjusted evaluation of symptom progression, enabling more precise and personalized interventions over time. In addition, prolonged follow-up strengthens the therapeutic relationship, enhancing the effects of interventions and promoting greater patient adaptation to the condition, which may result in significant improvement in quality of life and in the ability to cope with tinnitus more resiliently^(38-42)^.

Overall, it can be predicted that auditory training, in its different forms of stimulation, may promote positive outcomes in individuals with tinnitus disorder, corroborating the scientific literature^(43,44)^. Multisensory perceptual training methods, involving auditory and cognitive activities, are more effective in promoting positive improvements in neural connectivity, which is also consistent with the specialized literature^(1)^.

Furthermore, the present study had some limitations, mainly related to sample size and differences in sample arrangement among groups due to participant loss throughout the study. In addition, the lack of an objective evaluation for neural measurement, through auditory evoked potentials, of the proposed interventions should also be highlighted as a limitation of the study, considering that the literature demonstrates that neural modifications tend to occur before behavioral responses.

Finally, this study provides important evidence for the literature, indicating that neuroplastic modifications in the central auditory pathway associated with auditory training resulting from musical auditory training and cognitive auditory training interventions tend to improve the perception of tinnitus disorder. Multisensory intervention involving auditory and cognitive tasks was more effective in reducing symptom perception for the participants of this study. Furthermore, the importance of patient support as a means of reducing stress associated with the symptom and, consequently, improving perception should be considered.

## CONCLUSION

This study allowed the identification of improvement in the perception of tinnitus disorder in the three interventions performed. Cognitive auditory training, musical auditory training, and placebo demonstrated improvement in annoyance perception and quality of life in individuals with tinnitus disorder. Cognitive auditory training also demonstrated improvement in relation to tinnitus loudness, suggesting that it is an option that promotes greater benefits due to multisensory stimulation.
