# Supplementary material for: Musical auditory training and cognitive auditory training: effects on the perception of tinnitus disorder
Source: Codas. 2026 Apr 3;38(2):e20250145. doi: 10.1590/2317-1782/e20250145en (PMC13075834; doi:10.1590/2317-1782/e20250145en)
Supplement: Translation

# Translation: Treinamento auditivo musical e treinamento auditivo cognitivo: efeitos na percepção do transtorno do zumbido

**DOI:** 10.1590/2317-1782/e20250145pt

**Authors:** Christine Grellmann Schumacher, Brenda Larissa de Souza da Silva, Roberta Liberalesso, Katya Guglielmi Marcondes Freire, Michele Vargas Garcia, Dayane Domeneghini Didoné

**Affiliations:** 1 Departamento de Fonoaudiologia, Centro de Ciências da Saúde, Universidade Federal de Santa Maria – UFSM - Santa Maria (RS), Brasil.

**Keywords:** Zumbido, Percepção Auditiva, Audição, Ensaio Clínico Controlado, Potenciais Evocados Auditivos

## Abstract

**Objetivo:**

Verificar os efeitos do Treinamento Auditivo Cognitivo (TAC) e do Treinamento Auditivo Musical (TAM) na autopercepção dos sujeitos com transtorno do zumbido na população adulta.

**Método:**

Estudo de caráter clínico comparativo preliminar, de intervenção e longitudinal. Foi aprovado pelo Comitê de Ética em Pesquisa da Universidade Federal de Santa Maria sob o número 5.746.241. A pesquisa contou com a participação de 29 adultos de ambos os sexos, com transtorno de zumbido e com resultados normais na avaliação audiológica básica. Os participantes foram divididos em três grupos: 13 indivíduos no grupo que recebeu o TAC, oito no grupo que recebeu o TAM e oito no grupo de intervenção placebo. Foram analisadas as pontuações de Incômodo e Volume na Escala Visual Analógica (EVA I e EVA V) e no Tinnitus Handicap Inventory (THI), antes e após as intervenções. Utilizou-se o teste de Wilcoxon para a comparação dos resultados pré e pós-intervenção, adotando-se um nível de significância de 5%.

**Resultados:**

No grupo TAC, observou-se diferença estatisticamente significante para as variáveis EVA I, EVA V e THI. No grupo TAM, as diferenças significativas foram verificadas para EVA I e THI. Já no grupo placebo, identificou-se diferença estatisticamente significante para EVA I e THI.

**Conclusão:**

Observou-se melhora na percepção do sintoma nos três grupos. No entanto, o TAC demonstrou maior efetividade, promovendo mudanças em todos os aspectos avaliados.

## INTRODUÇÃO

Pesquisas sobre a efetividade de diferentes intervenções para o zumbido são importantes, visto a escassez de diferentes protocolos de tratamento para reorganização das áreas cerebrais auditivas e não auditivas e diminuição da percepção do sintoma^(1)^.

O zumbido está diretamente relacionado a alterações na atividade neuronal de vias auditivas centrais^(2,3)^. A base neurobiológica desse sintoma é definida por uma atividade espontânea anormal contínua, caracterizada pela desorganização das vias neurais do sistema auditivo central^(3)^. Além disso, há uma desorganização das áreas cerebrais não auditivas, incluindo a área parietal frontal responsável pela consciência e atenção; na rede neural relacionada às emoções, que consiste no córtex cingulado anterior, na ínsula anterior e na amígdala; no hipocampo e a área parahipocampal, que reflete a atividade dos mecanismos de memória e cognição, as quais também estão associadas a persistência da percepção do zumbido, gerando ansiedade, angústia e sofrimento^(2)^.

O zumbido é um sintoma auditivo multifatorial, caracterizado pela percepção de um som na ausência de estímulos acústicos externos, sendo que a percepção do sujeito ocorre na cabeça ou nas orelhas^(1)^. Estima-se que este sintoma afeta cerca de 21% da população adulta, e que 1% a 3% dos sujeitos apresentam impacto significativo em suas atividades cotidianas^(4,5)^.

A identificação de um sofrimento decorrente da percepção do zumbido leva a definição do termo “transtorno do zumbido”, que pode ser descrito como a percepção consciente de um som tonal ou composto, associado a disfunção emocional, alteração cognitiva e/ou excitação autonômica, levando a alterações comportamentais e incapacidade funcional^(6)^. Além deste impacto nas atividades diárias, a literatura relata a repercussão do sintoma nas atividades sociais, emocionais e profissionais, o que causa repercussão negativa na qualidade de vida desses sujeitos e, consequentemente, maior percepção e incômodo frente ao zumbido^(7)^.

Sadeghijam et al.^(8)^ descreveram a teoria do caos, em que o transtorno do zumbido é um fator resultante de mudanças no funcionamento dinâmico e não linear do sistema auditivo central. Sendo assim, qualquer alteração neural, decorrente do desequilíbrio entre a excitabilidade e o mecanismo de inibição ou da diminuição do *input* auditivo resultará em um mecanismo compensatório, convertendo em uma amplificação da atividade neural espontânea e síncrona^(8)^.

Uma das alternativas para reorganizar as vias auditivas neurais é por meio do treinamento auditivo^(9,10)^, caracterizado como uma forma de terapia composta por um conjunto de tarefas que buscam reorganizar o funcionamento do sistema auditivo central por meio da neuroplasticidade positiva. A neuroplasticidade auditiva é a capacidade do sistema nervoso central de reorganizar e adaptar suas conexões neurais em resposta a estímulos sonoros e experiências auditivas, promovendo mudanças funcionais nas áreas relacionadas à audição^(11)^.

Sujeitos com transtorno do zumbido podem apresentar uma disfunção neuroplástica devido a deaferentação das regiões centrais^(12,13)^, sendo que essas alterações podem ser restabelecidas com o treinamento auditivo, o qual reorganiza a via auditiva central por meio de exercícios acústicos que promovem a maior sincronia neural devido ao mecanismo da neuroplasticidade^(1)^.

Pesquisadores^(1,14)^ descrevem que o treinamento auditivo pode ser uma opção para a redução da percepção e incômodo do zumbido. Ainda, tarefas auditivo-cognitivas são descritas como essenciais nos casos de zumbido, conforme apontado por uma revisão sistemática^(1)^, que identificou que fatores atencionais e multissensoriais apresentaram melhora dos resultados.

Existem diferentes propostas de treinamento auditivo, sendo uma delas o Treinamento Auditivo Musical (TAM), definido como um instrumento que pode gerar mudanças nas vias auditivas, tanto estruturais quanto funcionais, e uma melhora das habilidades auditivas e do desempenho do indivíduo frente a diferentes eventos acústicos, sendo relacionado a capacidade de reorganização das vias neurais^(15)^. As evidências científicas demonstram que esta é uma intervenção para ser realizada quando há alterações no processamento auditivo, mas esta técnica pode promover a reorganização neural necessária no transtorno do zumbido^(15)^. Contudo, ainda não há estudos que comprovem sua efetividade frente à percepção do sintoma na população com transtorno do zumbido.

Já o Treinamento Auditivo Cognitivo (TAC), pode ser uma proposta de intervenção na diminuição da percepção do zumbido, uma vez que visa estimular as habilidades auditivas e cognitivas, como a atenção, memória, figura-fundo para sons verbais, ordenação e resolução temporal, fechamento auditivo, funções executivas e praxias motoras^(16)^. Estudos têm evidenciado que estimular as habilidades cognitivas nos casos de transtorno do zumbido pode ser fundamental para redução da percepção do sintoma, já que as consequências do zumbido no sistema nervoso central envolvem uma reorganização plástica que abrangem áreas auditivas e não auditivas do sistema nervoso central e que o aprendizado perceptual multissensorial tende a ser mais consistente do que o aprendizado unimodal^(17)^.

Dessa forma, na tentativa de contribuir com a lacuna científica referente às limitadas formas de intervenção e sua efetividade para o transtorno do zumbido, o presente estudo buscou verificar os efeitos do Treinamento Auditivo Cognitivo (TAC) e do Treinamento Auditivo Musical (TAM) na autopercepção dos sujeitos com transtorno do zumbido na população adulta.

## MÉTODO

Trata-se de um estudo de caráter clínico comparativo, preliminar, de intervenção e longitudinal. O estudo foi aprovado pelo Comitê de Ética em Pesquisa da Universidade Federal de Santa Maria, sob parecer nº 5.746.241. Antes de iniciar a pesquisa, todos os participantes assinaram o Termo de Consentimento Livre e Esclarecido (TCLE). O estudo foi realizado no Ambulatório de Audiologia da Universidade citada. A pesquisa foi divulgada nas redes sociais, sendo que a seleção dos sujeitos ocorreu de forma aleatória, não-probabilística.

Durante a fase de recrutamento dos sujeitos, estipulou-se os seguintes critérios de elegibilidade: sujeitos de ambos os sexos, ter idade entre 18 e 55 anos, ter queixa de transtorno do zumbido subjetivo uni ou bilateral, com percepção do sintoma de no mínimo seis meses^(18)^, não estar realizando intervenção ou tratamento farmacológico para o zumbido e não possuir diagnóstico de doenças neurológicas ou psiquiátricas. Para os sujeitos que se enquadraram no recrutamento, estabeleceu-se os seguintes critérios de inclusão:

- Limiares auditivos dentro dos padrões de normalidade ou perda auditiva sensorioneural de grau leve de acordo com a Organização Mundial da Saúde^(19)^;
- Curva timpanométrica do tipo A, de acordo com a classificação de Jerger^(20,21)^;
- Reflexos acústicos estapedianos contralaterais presentes e em níveis normais^(22,23)^;
  - Nota de incômodo da Escala Visual Analógica (EVA) de no mínimo 4, indicando incômodo moderado do sintoma, pré intervenção^(24)^.

Foram considerados os seguintes critérios de exclusão:

- Não concluir alguma das etapas propostas para o estudo.

### Procedimentos de composição da amostra

Inicialmente, os voluntários foram submetidos a uma anamnese baseada no *Clinical Practice Guideline: Tinnitus* ^(25,26)^ para compreender a história clínica. Os sujeitos foram questionados sobre a audição, histórico pregresso do zumbido e sobre comprometimento neurológico ou tratamentos psiquiátricos.

Posteriormente, foram submetidos a inspeção visual do meato acústico externo, com otoscópio da marca *Mikatos*, com o objetivo de verificar possíveis impedimentos para realização dos demais procedimentos audiológicos.

Todos os voluntários realizaram a avaliação audiológica básica, composta por audiometria tonal liminar e logoaudiometria, em cabina acusticamente tratada, com audiômetro AD229e da marca *Interacoustics e* fones supra-aurais do tipo TDH-39, da marca *Telephonics*.

Os limiares auditivos por via aérea foram pesquisados nas frequências de 250Hz até 8kHz, bilateralmente, por meio da técnica descendente-ascendente. Considerou-se como critério de normalidade limiares menores que 20dBNA, de acordo com a média quadritonal de 500Hz, 1kHz, 2kHz e 4kHz, proposto pela OMS^(19)^.

As medidas de imitância acústica foram realizadas no equipamento AT 235 da marca Interacoustics, com o uso do fone TDH-39 da marca *Telephonics*. As curvas timpanométricas e os reflexos acústicos estapedianos contralaterais (frequências de 500, 1000, 2000 e 4000Hz) foram pesquisadas com o objetivo de averiguar a integridade da orelha média e do arco reflexo. A análise dos resultados da curva timpanométrica foi realizada de acordo com Jerger, Jerger e Mauldin^(21)^e dos reflexos acústicos estapedianos contralaterais conforme Gelfand^(22)^ e Jerger e Jerger^(23)^.

### Procedimentos de pesquisa

Posteriormente às avaliações audiológicas descritas, os sujeitos foram submetidos à avaliação da mensuração do incômodo e volume do zumbido. Ressalta-se que todos os equipamentos utilizados foram calibrados em março de 2023, acompanhando a data da coleta de dados do presente estudo. Para tal foram utilizados os instrumentos Escala Visual Analógica (EVA) e *Tinnitus Handicap Inventory* (THI).

A análise da autopercepção do transtorno do zumbido é um fator importante, constituindo uma abordagem complementar para mensurar o impacto do zumbido na vida do paciente pré e pós intervenção^(27)^. Há diversos instrumentos que podem ser utilizados para mensurar e monitorar a percepção do indivíduo sobre o sintoma, dentre os quais destacam-se a Escala Visual-Analógica (EVA) e o questionário *Tinnitus Handicap Inventory* (THI)^(28)^.

Desta forma, pode-se mensurar de forma subjetiva o nível de desconforto dos participantes sobre o sintoma do zumbido. Ambos os procedimentos, THI e EVA, foram utilizados na avaliação e após duas semanas após finalização da intervenção.

A quantificação do grau de incômodo e de volume foi definida por meio da escala EVA, uma escala de autopercepção, a qual foi apresentada por meio de uma figura com numeração de zero a dez. Os voluntários foram questionados quanto à percepção subjetiva do grau de incômodo e volume e orientados a apontar o número que mais se adequasse a sua perceção, sendo que para ambas as situações a nota zero significou nenhum incômodo e zumbido sem volume e dez incômodo severo e volume muito alto^(24)^. Ademais, o critério de inclusão de nota de no mínimo quatro pontos indicando incômodo moderado, caracteriza a percepção do sujeito como transtorno do zumbido.

Já o THI, é um questionário composto por 25 questões referentes à vida social e pessoal do indivíduo, as quais estão diretamente relacionadas às mudanças negativas que o zumbido provoca no cotidiano e sua interferência na qualidade de vida destes sujeitos. As perguntas foram aplicadas de forma oral, com possibilidade de resposta de “Não”, “Às vezes” e “Sim”, com pontuação respectiva de zero, dois e quatro pontos. Para mensurar o incômodo do sintoma utilizou-se a somatória das questões, sendo que quanto menor o valor menor o incômodo percebido^(20)^.

### Procedimentos de intervenção

Em relação aos procedimentos de intervenção, os sujeitos foram alocados aleatoriamente em três grupos, de acordo com a ordem de chegada para o atendimento: Grupo TAC, que recebeu a intervenção por meio do TAC; Grupo TAM, grupo que recebeu a intervenção por meio do TAM e; Grupo placebo, grupo que recebeu a intervenção placebo. Todas as intervenções ocorreram no formato presencial, aplicadas por um pesquisador.

No TAC, foi utilizado o protocolo proposto por Schumacher et al.^(29)^ e foram aplicadas seis sessões de treinamento de habilidades auditivas e cognitivas, semanalmente, totalizando seis semanas, ou seja, uma sessão por semana. Estas tiveram duração de 45 a 50 minutos. Ao longo da intervenção todas as estratégias buscaram estimular habilidades auditivas e aspectos cognitivos dos participantes, tais como: figura fundo para sons verbais e atenção seletiva; atenção; ordenação temporal para duração; atenção seletiva, funções executivas, praxia motora; discriminação auditiva para sons não verbais; praxia construtiva; linguagem; processamento do discurso; ordenação temporal para frequência, memória. No Quadro 1 é possível visualizar o protocolo de TAC utilizado no presente estudo.

**Quadro 1:** Protocolo de Treinamento Auditivo Cognitivo utilizado no presente estudo, considerando as habilidades auditivas e cognitivas estimuladas em cada sessão

**Sessão**	**Habilidade Estimulada**
1º	- Atenção;
	- Auditivas: figura fundo para sons
	- Verbais e atenção seletiva;
	- Memória;
	- Ordenação temporal para duração;
	- Ordenação temporal;
2º	- Atenção;
	- Funções executivas;
	- Praxia motora;
	- Memória;
	- Discriminação;
	- Auditiva para sons não verbais;
3º	- Atenção;
	- Memória;
	- Funções executivas;
	- Praxia construtiva;
	- Discriminação auditiva para sons não verbais;
4º	- Linguagem;
	- Memória;
	- Figura fundo para sons verbais e atenção seletiva;
	- Atenção;
	- Processamento do discurso;
	- Ordenação temporal para frequência;
	- Discriminação auditiva para sons
	não verbais;
5º	- Figura fundo para sons verbais;
	- Atenção;
	- Discriminação auditiva;
	- Memória;
	- Ordenação auditiva;
	- Fechamento auditivo;
6º	- Memória;
	- Ordenação temporal;
	- Resolução temporal;

O protocolo utilizado para aplicação do TAM foi proposto por Freire^(15)^. Foram realizadas oito sessões, com variação de tempo de 45 a 50 minutos por sessão. Durante esse período de quatro semanas, foram estimuladas as habilidades auditivas de figura -fundo para sons instrumentais, figura-fundo para sons sequenciais, escuta direcionada, ordenação temporal para duração e frequência, ritmo, fechamento auditivo e memória audiovisual. Ao início de cada sessão, os participantes foram orientados quanto às atividades que seriam realizadas no dia. O treinamento auditivo foi realizado com uso de fones supra-aurais, em intensidade acusticamente confortável. O Quadro 2 especifica o protocolo de TAM utilizado no presente estudo.

**Quadro 2:** Protocolo de Treinamento Auditivo Musical utilizado no presente estudo, considerando as habilidades auditivas estimuladas em cada sessão

**Sessão**	**Habilidade Estimulada**
1º	Figura fundo para sons instrumentais
2º	Figura fundo para sons sequenciais
3º	Escuta direcionada
4º	Ordenação temporal para duração
5º	Ordenação temporal para frequência
6º	Ritmo
7º	Fechamento auditivo
8º	Memória audiovisual

O protocolo utilizado para o grupo placebo considerou a exposição a filmes e músicas clássicas em ordem aleatória e apresentados nas diferentes sessões. Os participantes foram orientados a prestar atenção visualmente ao filme (apresentado sem som) e auditivamente à música. Os filmes apresentados foram os seguintes: Cirque Du Soleil, intitulado “A Jornada do Homem”; Coleção do Chaplin com os seguintes filmes: “Tempos Modernos”, “O grande ditador”, “Em busca do Ouro” e “Luzes da Ribalta”. Ressalta-se que os filmes escolhidos foram de obras cinematográficas que independem do diálogo dos atores para que o telespectador possa entender. A música apresentada foi Sonata para dois pianos em Ré maior, K448, de Mozart, sem mascarar o volume do zumbido. O protocolo do grupo placebo contou com oito sessões, as quais foram realizadas duas vezes por semana, com duração de no mínimo 45 a 50 minutos.

Após duas semanas das intervenções realizadas, os sujeitos responderam novamente ao questionário THI e EVA, visando mensurar as possíveis mudanças na percepção do sintoma com as intervenções realizadas. Os resultados do THI e EVA foram comparados entre si, de acordo com a distribuição dos indivíduos nos grupos descritos.

No total 95 sujeitos se candidataram para participar da pesquisa. Contudo, 11 sujeitos não foram recrutados por apresentar doenças neurológicas e/ou psiquiátricas com diagnóstico prévio e 13 por já estarem em algum tratamento para o zumbido. Dos 71 sujeitos que realizaram as avaliações para composição da amostra, 14 foram excluídos por apresentar otite e/ou disfunção da tuba auditiva e 18 por terem perda auditiva de grau moderado a perda auditiva completa. Ainda, 10 sujeitos foram excluídos por não concluírem todas as etapas do estudo.

Desse modo, a casuística total do estudo foi composta por 29 sujeitos distribuídos de forma aleatória nos três grupos. Os grupos foram compostos por 13 indivíduos no grupo TAC, 8 no grupo TAM e 8 no grupo placebo. A diferença da distribuição dos sujeitos entre os grupos ocorreu pela perda de seguimento de sujeitos no grupo que recebeu a intervenção por meio do TAM e grupo placebo.

Destaca-se que adotou-se delineamento duplo cego para realização da pesquisa, no qual os sujeitos não sabiam qual modelo de intervenção estariam recebendo, bem como, foi designado um pesquisador diferente para cada etapa da pesquisa, avaliação, intervenção e reabilitação. Após a intervenção placebo, foi ofertado seguimento de tratamento para os sujeitos, por meio do TAM ou do TAC, a critério de escolha do sujeito de acordo com sua afinidade pela proposta de cada uma das intervenções.

Os dados referente a escala EVA e ao questionário THI pré e pós intervenção foram alocados em planilha no *Excel* e as análises estatísticas foram realizadas no Programa SPSS, versão 30.0 por um profissional da área. Inicialmente foi utilizado o teste Shapiro-Wilk para verificar a normalidade dos dados e, consequentemente, escolha do teste estatístico. A análise entre os grupos foi realizada por meio do teste Kruskal Wallis, e nos casos de diferença estatisticamente significante, os dados foram comparados em pares. A comparação entre pares foi realizada por meio do teste post-hoc Bonferroni. As comparações intra grupos foram realizadas por meio do teste *Wilcoxon*. Considerou-se o nível de significância de 5% para todas as análises realizadas.

## RESULTADOS

Os grupos foram considerados equivalentes por meio da comparação estatística, sem significância. A amostra do estudo foi composta por três grupos com as seguintes distribuições de sexo: TAC (6 homens e 7 mulheres), TAM (3 homens e 5 mulheres) e grupo placebo (3 homens e 5 mulheres), sem diferença estatisticamente significativa entre eles (p-valor= 0,333), conforme análise pelo teste exato de Fisher. A média de escolaridade, expressa em anos de estudo, foi de 12,69 (mínimo de 12 e máximo de 15) no grupo TAC, 13,5 (mínimo de 12 e máximo de 15) no grupo TAM e 13,13 (mínimo de 12 e máximo de 15) no grupo placebo sem diferença estatisticamente significativa (p-valor= 0,454), conforme o teste de Kruskal-Wallis. A média de idade foi de 29,64 anos (mínimo de 22 e máximo de 49) no grupo TAC, 37 anos (mínimo de 22 e máximo de 53) no grupo TAM e 31 anos (mínimo de 20 e máximo de 53) no grupo placebo, também sem diferença estatisticamente significativa entre os grupos (p-valor= 0,379), segundo o teste de Kruskal-Wallis.

Os dados da escala EVA para incômodo (EVA I), EVA volume (EVA V) e THI pré intervenção foram comparados entre os grupos. Verificou-se homogeneidade entre os grupos para EVA V e EVA I (Tabela 1). Contudo, a comparação entre os três grupos evidenciou diferenças para o THI (p=0,006). A comparação em pares (método pairwise) mostrou diferenças entre o grupo TAM e placebo (p=0,004) para o THI pré intervenção, com valores menores para o grupo TAM. Para a comparação entre TAM e TAC (p=0,344) e TAC e placebo (p=0,143) não houve diferença estatisticamente significante.

**Tabela 1:** Comparação da escala EVA V, EVA I e THI pré intervenção entre os grupos

	Grupo	Mínimo	Máximo	Média	Mediana	P-valor
EVA I pré	TAM	4	9	7	7,5	0,900
	TAC	3	10	6,85	8	
	Placebo	4	10	6,38	6,50	
	TAM	5	8	6,63	7	0,560
EVA V pré	TAC	4	10	7,15	8	
	Placebo	4	9	6,38	6,50	
	TAM	20	58	34	31	0,006
THI pré	TAC	22	80	47,23	40	
	Placebo	36	94	66	66	

**Legenda:** EVA I: Escala Visual Analógica para Incomodo; EVA V: Escala Visual Analógica para Volume; THI: Tinnitus Handicap Inventory: Para análise estatística foi utilizado o teste Kruskal Wallis

Os dados da escala EVA I, EVA V e THI também foram comparados entre os grupos, pós intervenção (Tabela 2), sendo constatada diferença estatisticamente significante para todas as variáveis entre os três grupos.

**Tabela 2:** Comparação da escala EVA I, EVA V e THI pós intervenção e entre os grupos

	Grupo	Mínimo	Máximo	Média	Mediana	P-valor
EVA I pós	TAM	1	8	4,38	4,50	0,040
	TAC	0	7	2,77	3,00	
	Placebo	1	8	4,38	4,50	
	TAM	3	8	5,63	5,50	0,003
EVA V pós	TAC	0	6	2,92	3,00	
	Placebo	3	8	5,63	5,50	
	TAM	2	32	18,25	18,00	0,015
THI pós	TAC	4	65	21	16,00	
	Placebo	19	58	39,38	39,00	

**Legenda:** EVA I: Escala Visual Analógica para Incomodo; EVA V: Escala Visual Analógica para Volume; THI: Tinnitus Handicap Inventory: Para análise estatística foi utilizado o teste Kruskal Wallis

A comparação em pares (método pairwise) mostrou diferenças para EVA I (p=0,050) e para EVA V (p=0,021) entre os grupos TAC e placebo, sendo os melhores resultados para o grupo TAC, evidenciado pela maior redução dos valores. Para EVA I, as demais comparações post hoc não evidenciaram diferenças (TAC e TAM, p=0,195; TAM e placebo, p=1,000).

Para a escala EVA V também foi evidenciada diferença estatisticamente significante entre os grupos TAM e TAC (método pairwise) (p=0,011) e TAC e placebo (p=0,021), sendo os resultados melhores para o grupo TAC (Tabela 2). A comparação entre TAC e TAM não evidenciou diferenças (p=1,000).

Para o questionário THI, verificou-se diferença estatisticamente significativa na comparação dos três grupos no momento pós intervenção (p=0,015). A análise em pares (método pairwise) evidenciou diferenças entre os grupos TAM e placebo (p=0,047), com resultados melhores para o grupo TAM e diferenças entre TAC e placebo (p=0,023), com resultados melhores para o grupo TAC. Na comparação entre TAC e TAM não foi evidenciada diferença estatisticamente significante (p=1,000) (Tabela 2).

Considerando as comparações intra grupos, na Tabela 3 é possível visualizar os valores da escala EVA para incômodo (EVA I), EVA volume (EVA V) e THI pré e pós intervenção para o grupo TAC. Observou-se diferença estatisticamente significante para todas as variáveis analisadas, sendo os resultados melhores após a intervenção com o TAC.

**Tabela 3:** Dados da escala EVA para incômodo, volume e resultado total do questionário THI para o grupo que recebeu a intervenção por meio do TAC

	Média	Mediana	Mínimo	Máximo	DP	p-valor
EVA I pré	6,85	8,00	4	10	2,23	0,002
EVA I pós	2,77	3,00	0	7	1,64	
EVA V pré	7,15	8,00	4	10	2,11	0,002
EVA V pós	2,92	3,00	0	6	1,55	
THI pré	47,23	40,00	22	80	18,61	0,001
THI pós	21,00	16,00	4	65	17,95	

**Legenda:** EVA I: Escala Visual Analógica para Incomodo; EVA V: Escala Visual Analógica para Volume; THI: Tinnitus Handicap Inventory; DP: desvio padrão. Para análise estatística foi utilizado o teste de *Wilcoxon*

Na Tabela 4 estão descritos os valores do grupo que recebeu TAM, com diferença estatisticamente significante para EVA I e THI, sendo todos os resultados melhores após a intervenção com o TAM.

**Tabela 4:** Dados da escala EVA para incômodo, volume e resultado total do questionário THI para o grupo que recebeu a intervenção por meio do TAM

	Média	Mediana	Mínimo	Máximo	DP	p-valor
EVA I pré	7,00	7,50	4	9	1,85	0,028
EVA I pós	4,38	4,50	1	8	2,13	
EVA V pré	6,63	7,00	5	8	0,91	0,102
EVA V pós	5,35	5,50	3	8	1,68	
THI pré	34,00	31,00	20	58	13,93	0,001
THI pós	18,25	18,00	2	32	11,73	

**Legenda:** EVA I: Escala Visual Analógica para Incomodo; EVA V: Escala Visual Analógica para Volume; THI: Tinnitus Handicap Inventory; DP: desvio padrão. Para análise estatística foi utilizado o teste de *Wilcoxon*

Na Tabela 5, estão descritos os resultados pré e pós intervenção para o grupo placebo, sendo observada diferença estatisticamente significante para EVA I e THI. Todos os resultados apontam valores melhores das escalas no momento pós.

**Tabela 5:** Dados da escala EVA para incômodo, volume e resultado total do questionário THI para o grupo que recebeu a intervenção placebo

	Média	Mediana	Mínimo	Máximo	DP	p-valor
EVA I pré	6,62	6,5	4	10	2,13	0,027
EVA I pós	4,88	5,0	1	8	2,29	
EVA V pré	6,38	6,5	4	9	1,68	0,053
EVA V pós	5,50	5,5	3	8	2,07	
THI pré	66,00	66,00	36	94	16,21	0,012
THI pós	39,38	39,00	19	58	14,41	

**Legenda:** EVA I: Escala Visual Analógica para Incomodo; EVA V: Escala Visual Analógica para Volume; THI: Tinnitus Handicap Inventory; DP: desvio padrão. Para análise estatística foi utilizado o teste de *Wilcoxon*

## DISCUSSÃO

Este estudo abordou dois tipos de intervenção por meio da estimulação de habilidades auditivas, as quais foram somadas a intervenções cognitivas e musicais, por meio do TAC, do TAM e de um grupo placebo, respectivamente. Estas, quando analisadas, demonstraram uma melhora da percepção do incômodo (EVA I) e da qualidade de vida (THI), sugerindo que todas as intervenções propostas modificaram a percepção do sintoma.

Na análise dos dados pré intervenção, pode-se constatar que os dados foram homogêneos para EVA I e EVA V em todos os grupos. Considerando a análise dos dados pós-intervenção, pode-se constatar que os resultados do grupo TAC foram melhores quando comparados com os grupos TAM e placebo. Esses resultados corroboram com uma pesquisa recente^(29)^ que também evidenciou melhora da percepção do transtorno do zumbido por meio do TAC, o que sugere que a estimulação auditiva e cognitiva promove benefícios na percepção do sintoma.

Para o questionário THI, as diferenças entre os grupos e pós intervenção foram constatadas entre os grupos TAM e placebo, com resultados melhores para o grupo TAM. Contudo, esses resultados devem ser interpretados com cautela, já que os dados do THI para esses grupos não foram considerados homogêneos na etapa pré intervenção. Pelo fato desse estudo ser preliminar, há que se considerar que essas diferenças pré intervenção podem ter relação com o reduzido tamanho amostral. Apesar disso, esses resultados são promissores, visto que o TAM é uma abordagem atual e permite reorganizar a via auditiva desses sujeitos e melhorar a percepção dos sintomas. Ressalta-se que não foram encontrados estudos na literatura científica para comparação desses dados com a literatura.

Também foram constatadas diferenças para o THI pós intervenção entre os grupos TAC e placebo, sendo os resultados melhores para o grupo TAC. A intervenção auditiva cognitiva tem se mostrado uma alternativa promissora para o tratamento do zumbido, visto que promove a reorganização das vias auditivas associada à cognição^(16,30)^.

As comparações intra grupos deste estudo, Tabelas 3, 4 e 5, vão ao encontro da revisão de escopo publicada recentemente^(1)^, na qual foram identificadas 15 pesquisas com abordagens de treinamento auditivo e cognitivo. Entre estas, apenas quatro não encontraram efeitos estatisticamente significantes após a intervenção. Isso sugere que o treinamento auditivo, em suas diferentes abordagens, é uma opção de reabilitação para o transtorno do zumbido.

Verificou-se que a intervenção com o TAC (Tabela 3) levou a melhorias significativas nos escores EVA I, EVA V e THI, indicando sua efetividade no tratamento da percepção do zumbido. Esses achados estão em consonância com estudos que empregaram treinamentos cognitivos^(30)^ e observaram melhorias em questionários sobre percepção do sintoma. Tal benefício pode ser associado à estimulação central promovida por esse treinamento, que estimula a neuroplasticidade de funções auditivas e cognitivas. Isso ocorre porque o zumbido induz uma reorganização plástica no sistema nervoso central, abrangendo tanto áreas auditivas quanto não auditivas, e o aprendizado da perceptual multissensorial se mostra mais duradouro e consistente em comparação ao aprendizado unimodal^(1)^.

Além disso, em um estudo sobre terapia cognitiva, incluindo a *Heidelberg Neuro-Music Therapy* aplicada por Krick et al.^(31)^, observou-se que o tratamento ajuda pacientes com zumbido crônico a deslocarem sua atenção da percepção auditiva fantasma para estímulos visuais. Essa mudança de foco pode envolver o giro angular, uma área do cérebro essencial para a orientação da atenção a estímulos sonoros mais significativos. Este achado fortalece o embasamento do TAC como uma opção de reabilitação, pois oferece uma estimulação auditiva e cognitiva.

Quanto aos achados do TAM (Tabela 4), obteve-se melhora dos resultados da escala EVA I e do questionário THI. Não foram constatados estudos sobre o efeito do TAM em pacientes com transtorno do zumbido, o que confere ineditismo a essa intervenção na população do presente estudo. Contudo, os benefícios verificados na presente pesquisa vão ao encontro de outro estudo com idosos usuários de amplificação sonora^(32)^. Apesar da pesquisa citada ter sido realizada com idosos com perda auditiva e usuários de dispositivos eletrônicos de amplificação, os autores verificaram benefícios do TAM na qualidade de vida, sintomas depressivos, aspectos cognitivos, resolução temporal e limitação em atividades de vida. A melhora desses sintomas após a estimulação com o TAM pode ser associada aos resultados do presente estudo, já que o impacto positivo do TAM nos aspectos citados influencia na percepção do zumbido. Isto pode refletir na melhora da qualidade de vida e incômodo do sintoma relatados pelos participantes deste estudo.

Apesar dos resultados satisfatórios na intervenção com o TAM, não foram observadas mudanças estatisticamente significantes para a escala EVA relacionada ao volume, sugerindo que o volume não interferiu na qualidade de vida da amostra estudada. Também pode-se ponderar que a percepção do volume não tenha melhorado devido ao fato do TAM não estimular tanto as habilidades cognitivas como acontece no TAC, já que a estimulação é focada em percepção musical e habilidades auditivas. Conforme já mencionado por outros estudos^(33,34)^, intervenções multissensoriais parecem ter maior efetividade para a redução da percepção do sintoma, fato que justifica os achados descritos.

Neste estudo, na intervenção placebo também verificou-se melhora significativas nos escores do THI e EVA I, evidenciando a relevância do acolhimento, favorecido pelo contato presencial e pela interação direta com os participantes durante as sessões. Cabe salientar que esta intervenção foi realizada em um formato duplo cego, assegurando que os participantes não tinham conhecimento se estavam recebendo o tratamento ativo ou o placebo. Estudos adicionais sustentam os efeitos benéficos da intervenção placebo, associando o acolhimento empático a uma atenuação dos sintomas relatados^(35-37)^.

A literatura destaca que o acolhimento que acontece na intervenção placebo e uma intervenção longitudinal desempenham papéis cruciais no tratamento de pacientes com transtorno de zumbido, contribuindo para uma abordagem terapêutica mais efetiva e humanizada. O acolhimento promove um ambiente seguro e de confiança, no qual o paciente sente-se compreendido e apoiado, o que pode reduzir o estresse associado ao zumbido e melhorar a adesão ao tratamento. Uma intervenção longitudinal, por sua vez, permite uma avaliação contínua e ajustada da evolução dos sintomas, possibilitando intervenções mais precisas e personalizadas ao longo do tempo. Além disso, o acompanhamento prolongado reforça a relação terapêutica, potencializando os efeitos das intervenções e promove maior adaptação do paciente à condição, o que pode resultar em uma melhora significativa na sua qualidade de vida e na sua capacidade de lidar com o zumbido de forma mais resiliente^(38-42)^.

De forma geral, pode-se predizer que o treinamento auditivo em suas diferentes estimulações, pode promover resultados positivos nos sujeitos com transtorno do zumbido, fato que corrobora com a literatura científica^(43,44)^. Métodos de treinamento perceptual multissensorial, envolvendo atividades auditivas e cognitivas são mais efetivos na melhora positiva da conectividade neural, fato que também está em consonância com a literatura especializada^(1)^.

Ademais, o estudo realizado apresentou algumas limitações, principalmente relacionadas ao tamanho da amostra e às diferenças do arranjo amostral entre os grupos devido à perda de sujeitos ao longo do estudo realizado. Ainda, a falta de uma avaliação objetiva para mensuração neural, através de potenciais evocados auditivos, das propostas de intervenção também deve ser pontuada como limitação do estudo, tendo em vista que a literatura demonstra que as modificações neurais tendem a ocorrer antes das respostas comportamentais.

Por fim, este estudo traz indícios importantes para a literatura, indicando que as modificações neuroplásticas em via auditiva central associadas ao treino auditivo decorrentes das intervenções do treinamento auditivo musical e treinamento auditivo cognitivo tendem a melhorar a percepção do transtorno do zumbido. A intervenção multissensorial envolvendo tarefas auditivas e cognitivas foi mais efetiva na redução da percepção do sintoma para os sujeitos desta pesquisa. Ainda, há que se considerar a importância do acolhimento como forma de redução do estresse associado ao sintoma e, consequentemente, melhora da percepção.

## CONCLUSÃO

Este estudo permitiu identificar a melhora da percepção do transtorno do zumbido nas três intervenções realizadas. O treinamento auditivo cognitivo, o treinamento auditivo musical e o placebo demonstraram melhora na percepção de incômodo e qualidade de vida em sujeitos com transtorno do zumbido. O treinamento auditivo cognitivo também evidenciou melhora em relação ao volume do zumbido, sugerindo ser uma opção que promove mais benefícios devido à estimulação multissensorial.
